# High-Fat Diet Consumption in Adolescence Induces Emotional Behavior Alterations and Hippocampal Neurogenesis Deficits Accompanied by Excessive Microglial Activation

**DOI:** 10.3390/ijms23158316

**Published:** 2022-07-27

**Authors:** Xiuting Yao, Chenxi Yang, Conghui Wang, Hong Li, Jingyi Zhao, Xiaomin Kang, Zhuodong Liu, Lingyan Chen, Xinyu Chen, Tianshu Pu, Qinyang Li, Lijie Liu

**Affiliations:** 1 Department of Physiology, Medical College, Southeast University, Nanjing 210009, China; 230208409@seu.edu.cn (X.Y.); 220203915@seu.edu.cn (C.Y.); 230218467@seu.edu.cn (C.W.); 2 School of Life Science and Technology, Southeast University, Nanjing 210096, China; 220193543@seu.edu.cn (H.L.); 220213919@seu.edu.cn (J.Z.); 220213913@seu.edu.cn (X.K.); 3 Medical College, Southeast University, Nanjing 210009, China; 213193742@seu.edu.cn (Z.L.); 213193299@seu.edu.cn (L.C.); 213191077@seu.edu.cn (X.C.); 213191044@seu.edu.cn (T.P.); 213191056@seu.edu.cn (Q.L.); 4 Jiangsu Provincial Key Laboratory of Critical Care Medicine, Department of Physiology, School of Medicine, Southeast University, Nanjing 210009, China

**Keywords:** high-fat diet, adolescence, hippocampal neurogenesis, microglia, depression, anxiety

## Abstract

Adolescence is a developmental epoch characterized by massive neural circuit remodeling; thus, the brain is particularly vulnerable to environmental influences during this period. Excessive high-fat diet (HFD) consumption, which is very common among adolescents, has long been recognized as a potent risk factor for multiple mood disorders, including depression and anxiety. However, the precise mechanisms underlying the influences of HFD consumption in adolescence on emotional health are far from clear. In the present study, C57BL/6 mice were fed a control diet (CD) or HFD for about 4 weeks from postnatal day (P) 28 to P60, spanning most of the adolescence period, and then subjected to behavioral assessments and histological examinations. HFD mice exhibited elevated levels of depression and anxiety, decreased hippocampal neurogenesis, and excessive microglial activation in the ventral hippocampus. Furthermore, in HFD-fed mice, microglia showed increased DCX^+^ inclusions, suggesting aberrant microglial engulfment of newborn neurons in HFD-fed adolescents. To our knowledge, this is the first observation suggesting that the negative effects of HFD consumption in adolescence on emotion and neuroplasticity may be attributed at least in part to aberrant microglial engulfment of nascent neurons, extending our understanding of the mechanism underlying HFD-related affective disorders in young people.

## 1. Introduction

Depression and anxiety are highly debilitating and often comorbid mental disorders that are associated with tremendous personal suffering and impose a burden on society and the economy [1]. Roughly half of all cases of lifetime mental disorders begin in childhood and adolescence. Depression and anxiety are the most common mood disorders experienced by adolescents [2,3]. Given the high disability rate and healthcare burden imposed by the alarming prevalence of mental disorders worldwide [4], further elucidating the pathophysiological mechanisms underlying the onset and development of mood disorders in adolescents and identifying potential therapeutic targets are important public health priorities.

As the global economy has rapidly developed, the consumption of a palatable high-fat diet (HFD) by adolescents has become common worldwide. Data from clinical and epidemiological studies suggest a strong association between excessive energy intake and emotional disorders [5,6,7]. Recently, converging evidence from different animal studies has suggested that HFD consumption during early life leads to the development of anxious and depressive-like behavior [8,9]. The fact that the neurocircuitry involved in depression and anxiety, such as the limbic system, is still undergoing maturation during adolescence partially explains the adolescent susceptibility to the negative emotional effects of environmental factors, such as an HFD [10,11,12,13]. However, the precise mechanisms underlying the detrimental effects of HFD consumption in adolescence on emotional health are far from clear.

The neural mechanisms underlying depression and anxiety remain incompletely understood; however, atrophy of the hippocampus, a key brain region related to emotion, is one of the most frequently reported structural neuroimaging findings in persons with depression and other neuropsychiatric disorders, indicating a central role for hippocampal neuroplasticity in the etiology and pathophysiology of emotional impairment [14,15]. Deficits in hippocampal neuroplasticity, such as neurogenesis impairment, are generally regarded as a major cause of hippocampal atrophy and dysfunction [16,17]. Hippocampal neurogenesis, a process through which additional granule cells are produced from progenitors located in the subgranular zone (SGZ) and added to the dentate gyrus (DG) throughout life, is modulated by internal and external factors, including environmental cues such as dietary fat intake [18,19]. The generation and integration of nascent neurons into established hippocampal neural circuitry are thought to play an important role in affective functions [20,21]. In rodents, unpredictable chronic mild stress decreases neurogenesis in the hippocampus, paralleling an increase in depressive-like behavior [22]. Another study using transgenic mice with neural stem cell-specific deletion of a proapoptotic gene demonstrated that increasing hippocampal neurogenesis is sufficient to alleviate anxiety- and depression-related behaviors [19]. It has also been found that antidepressant medications and therapies increase neurogenesis in depressed patients [20]. It has been demonstrated that the density of granule cells is increased and the level of hippocampal neurogenesis is elevated in adolescent rodents compared to adult rodents [23]. Given the role of hippocampal neurogenesis in psychiatric disorders in adults and the increase in neurogenesis in the adolescent brain, it is plausible that disruptions in neurogenesis during adolescence might be implicated in the development of various neuropsychiatric disorders, including depression and anxiety, in young people.

Microglia, macrophage-like immune cells in the brain parenchyma, are highly motile cells that dynamically survey the conditions of the brain and play integral roles in maintaining biochemical homeostasis throughout life and orchestrating the brain response to various internal and external stimuli [24]. In addition to playing these typical immunological roles, microglia have been revealed to be active players in complex neurodevelopment [25], acting as universal sensors and versatile modulators of hippocampal neurogenesis that can initiate and orchestrate context-dependent processes to contribute to the formation of functional neuronal connections and maintain an optimal neural network [26]. While close regulation of activity is necessary for healthy neurodevelopment, excessive or prolonged activation of microglia, especially in the hippocampus, during critical developmental periods, including adolescence, can have deleterious effects on brain function and neurobehavioral function [21,27,28,29]. However, whether and how microglia are involved in hippocampal neurogenesis deficits and emotional disorders associated with HFD consumption in adolescence remains largely speculative.

In the present study, we evaluated the effects of HFD consumption in adolescence on mood, hippocampal neurogenesis, microglial phenotype, and the potential roles of microglia in neuropsychiatric disorders related to HFD consumption in adolescence. Here, we found that HFD consumption in adolescence had a negative effect on affective behaviors and that hippocampal neurogenesis was associated with excessive microglial activation in the hippocampus. Furthermore, microglia in the ventral hippocampus showed significantly increased DCX^+^ inclusions in HFD-fed mice, suggesting increased microglial engulfment of newborn neurons after HFD consumption during adolescence. These results corroborate and extend previous findings, indicating that HFD-related mood disorders and hippocampal neurogenesis impairment in adolescents are attributed, at least partially, to excessive microglial phagocytosis of newborn cells.

## 2. Results

### 2.1. HFD Consumption in Adolescence Induces Depressive and Anxiety-like Behavior in Mice

**Figure 1:**
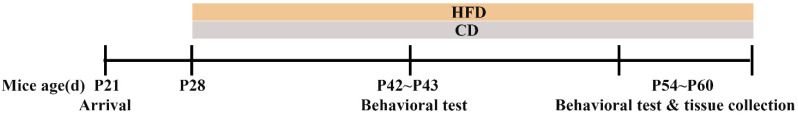
Experimental design showing the age of the animals, duration of dietary treatment, and timing of behavioral testing and tissue collection.

**Figure 2:**
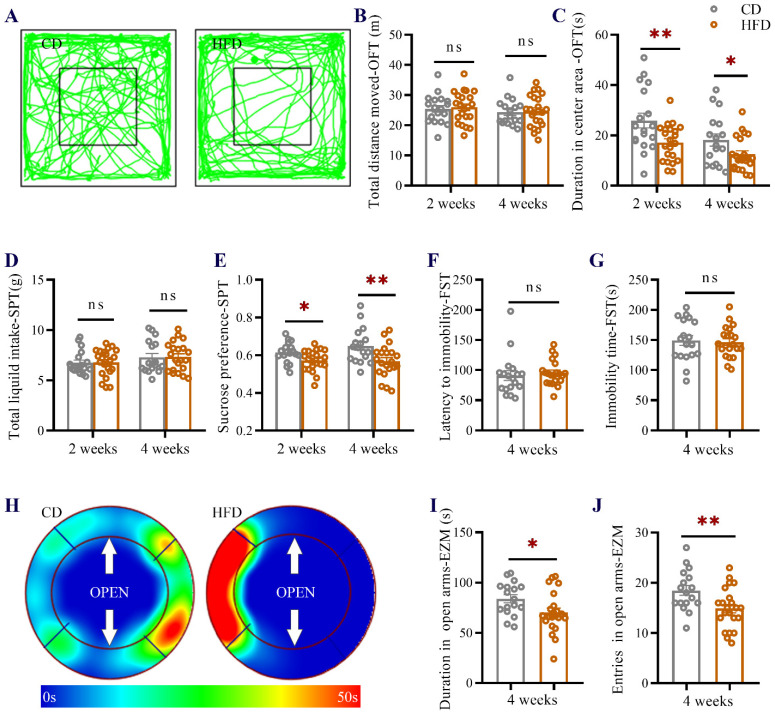
Effect of HFD consumption in adolescence on affective behavior in mice. (**A**) Movement paths (after 4 weeks of CD or HFD feeding), (**B**) total distance traveled, and (**C**) time spent in the center area in the OFT test for CD-fed and HFD-fed mice. (**D**) Total liquid intake and (**E**) sucrose preference ratio in the SPT. (**F**) Latency to immobility and (**G**) immobility time in the FST. (**H**) Heatmap, (**I**) duration spent in the open arms, and (**J**) the number of entries into the open arms in the EZM. The data are expressed as the mean ± standard error of the mean (SEM) (*n* = 17–23 mice per group). The data were analyzed using two-way ANOVA. When the HFD group was compared with the CD group, Student’s *t*-test was used. ns, not significant; * *p* < 0.05; ** *p* < 0.01.

To investigate the influence of HFD consumption in adolescence on emotional functions in mice, the sucrose preference test (SPT), open field test (OFT), elevated zero maze (EZM), and forced swim test (FST) were conducted after 2 weeks and 4 weeks of dietary treatment (behavioral test in Figure 1). As shown in Figure 2A–C, HFD consumption in adolescence (2 weeks and 4 weeks of HFD feeding) did not affect the total distance traveled in the OFT (Figure 2A,B) but significantly reduced the time in the central square (two-way analysis of variance (ANOVA), main effect of diet: F (1, 75) = 11.97, *p* = 0.0009, Figure 2C), indicating that HFD-fed mice exhibited increased anxiety-like behavior without impairment of athletic ability. Total liquid intake in the SPT was comparable between control diet (CD)- and HFD-fed (two-way ANOVA, main effect of diet: F (1, 74) = 0.0106, *p* = 0.9182, Figure 2D), while sucrose preference was significantly decreased in HFD-fed mice (two-way ANOVA, main effect of diet: F (1, 74) = 13.51, *p* = 0.0004, Figure 2E), indicating that anhedonia was induced after 2 weeks and 4 weeks of HFD feeding. In the FST, there was no significant difference between groups in the latency to immobility (Student’s *t*-test: *p* = 0.6051, Figure 2F) or immobility time (Student’s *t*-test: *p* = 0.7658, Figure 2G). In the EZM, HFD-fed mice showed significant increases in the amount of time spent in (Student’s *t*-test: *p* = 0.0287, Figure 2H,I) and the number of entries into (Student’s *t*-test: *p* = 0.0090, Figure 2J) the open arms, further suggesting that HFD consumption induced anxiety-like behavior in mice. Taken together, these data demonstrated that anxiety- and depression-like behavior was induced in HFD-fed adolescent mice.

### 2.2. HFD Consumption in Adolescence Decreases Hippocampal Neurogenesis, Especially in the Ventral Hippocampus

**Figure 3:**
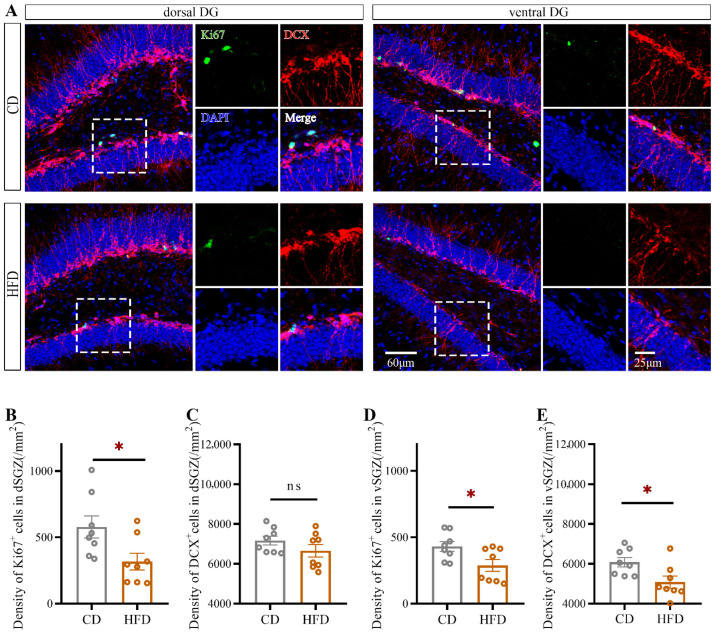
Effect of HFD consumption in adolescence on DCX and Ki67 expression in the mouse hippocampus. (**A**) Fluorescence staining of DCX^+^ and Ki67^+^ cells in the dorsal and ventral hippocampus of CD- and HFD-fed mice. Green, red and blue fluorescence represent Ki67, DCX, and DAPI, respectively. The numbers of Ki67^+^ and DCX^+^ cells in the dSGZ (**B**,**C**) and vSGZ (**D**,**E**) were counted. The data are expressed as the mean ± SEM (*n* = 8 mice per group). When the HFD group was compared with the CD group, the data were analyzed using Student’s *t*-test. ns, not significant; * *p* < 0.05.

To investigate the effect of HFD consumption in adolescence on hippocampal neurogenesis, we quantified the number of cells positive for Ki67 (a proliferating cell marker) and the number of cells positive for doublecortin (DCX, a marker of newly generated neurons within the last 2–3 weeks) in the dorsal and ventral hippocampus separately (Figure 3A). Compared with CD-fed mice, HFD-fed mice exhibited a significant reduction in the number of Ki67^+^ cells in both the dorsal SGZ (dSGZ, Student’s *t*-test: *p* = 0.0247, Figure 3B) and ventral SGZ (vSGZ, Student’s *t*-test: *p* = 0.0264, Figure 3D) and a significant reduction in the number of DCX^+^ cells in the vSGZ (Student’s *t*-test: *p* = 0.0195, Figure 3E).

**Figure 4:**
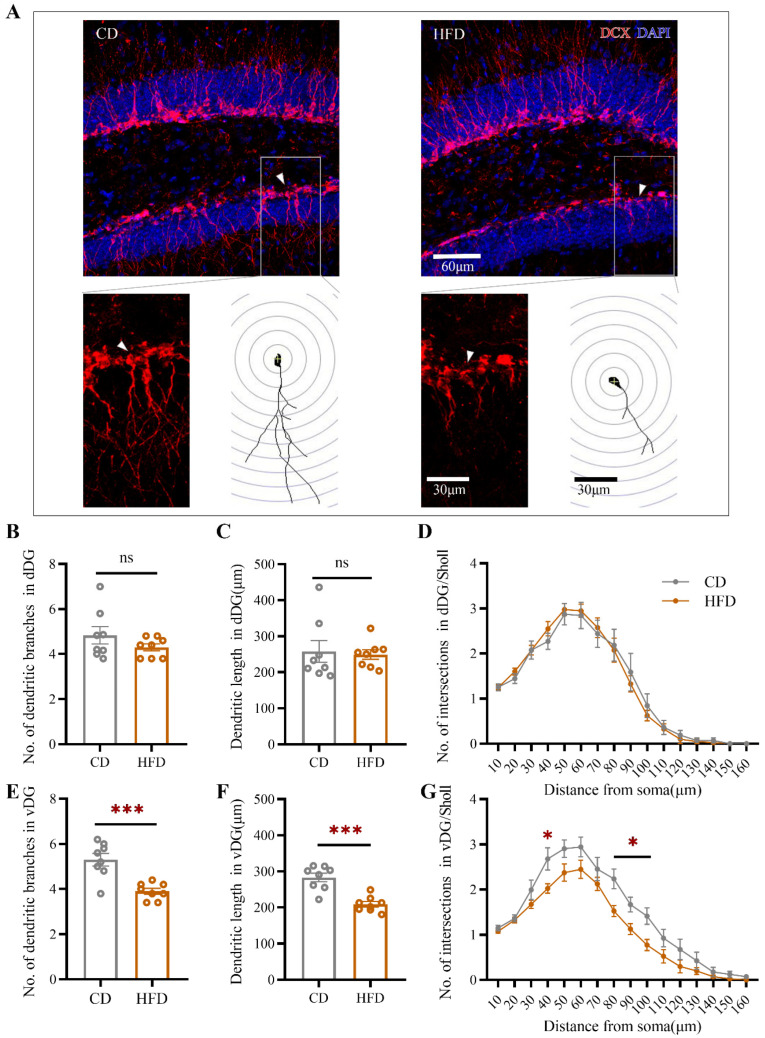
Effect of HFD consumption in adolescence on the morphology of DCX^+^ cells in the mouse hippocampus. (**A**) Fluorescence staining and traces of DCX^+^ cells in the ventral hippocampus of CD- and HFD-fed mice. Red and blue fluorescence represent DCX and DAPI, respectively. The total dendritic length, total number of dendritic branches, and number of intersections (Sholl analysis) of DCX^+^ cells in the dorsal dentate gyrus (dDG) (**B**–**D**) and vDG (**E**–**G**) were determined. The data are expressed as the mean ± SEM (*n* = 6 mice per group). When HFD group was compared with the CD group, the data were analyzed using Student’s *t*-test. ns, not significant; * *p* < 0.05; *** *p* < 0.001.

Next, we performed a morphological analysis of DCX^+^ cells in the dorsal and ventral hippocampus (for ventral hippocampus: Figure 4A). At least five cells from each animal were selected randomly and traced using ImageJ. As shown in Figure 4, for DCX^+^ cells, the total number of dendritic branches (Student’s *t*-test: *p* = 0.0004, Figure 4E), the total dendritic length (Student’s *t*-test: *p* = 0.0001, Figure 4F), and the number of dendritic intersections with concentric circles according to the Sholl analysis (Figure 4G) were decreased in the ventral hippocampus in HFD-fed mice, indicating atrophy of newborn neurons. No significant changes in these parameters were observed in the dorsal hippocampus (Figure 4B–D).

These data indicated that hippocampal neurogenesis was impaired after HFD consumption in adolescence and that newborn neurons in the ventral hippocampus were more susceptible than those in the dorsal hippocampus to HFD consumption in adolescence.

### 2.3. Impairment of Ventral Hippocampal Neurogenesis Is Significantly Related to Depression-like Behavior but Not to Anxiety-like Behavior

**Figure 5:**
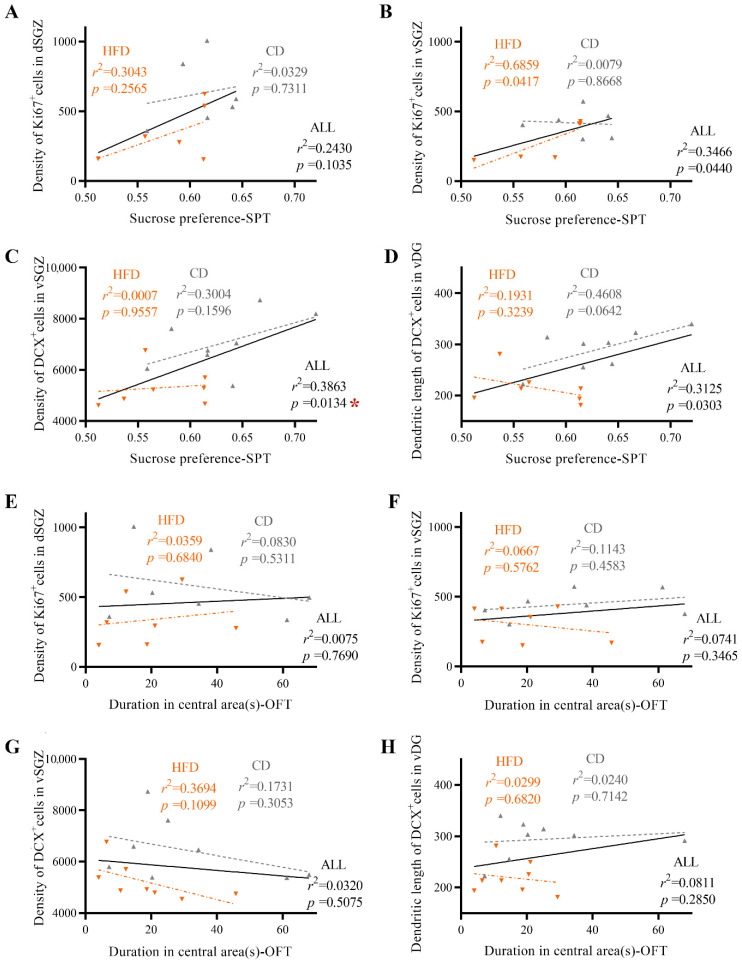
Linear regression analysis of hippocampal neurogenesis and depression-like and anxiety-like behavior. (**A**,**B**) Correlation between the density of Ki67^+^ cells in the dSGZ/vSGZ and sucrose preference in the SPT. (**C**) Correlation between the density of DCX^+^ cells in the vSGZ and sucrose preference in the SPT. (**D**) Correlation between the dendrite length of DCX^+^ cells in the vDG and sucrose preference in the SPT. (**E**,**F**) Correlation between the density of Ki67^+^ cells in the dSGZ/vSGZ and the time spent in the central area in the OFT. (**G**) Correlation between the density of DCX^+^ cells in the vSGZ and time spent in the central area in the OFT. (**H**) Correlation between the dendrite length of DCX^+^ cells in the vDG and the time spent in the central area in the OFT. Mice from both the CD group (represented as gray triangles) and HFD group (represented as orange triangles) are included (*n* = 6–8 mice per group). The solid black lines, gray dotted lines, and orange dotted lines indicate linear regression for mice fed a CD or HFD, mice fed a CD, and mice fed an HFD, respectively. * means statistical significance after Bonferroni correction.

To investigate whether the emotional behavior alterations of HFD-fed mice could be explained by the alterations in hippocampal neurogenesis, we examined the association between indexes of hippocampal neurogenesis and behavioral traits (only significantly altered indexes were analyzed). As illustrated in Figure 5A–H, when both the CD-fed group and HFD-fed group were considered, the density of Ki67^+^ cells (Figure 5B) and DCX^+^ cells (Figure 5C) in the vSGZ as well as the dendritic length of DCX^+^ cells (Figure 5D) in the ventral dentate gyrus (vDG) were significantly correlated with sucrose preference in the SPT (for density of Ki67^+^ cells: *r*^2^ = 0.3466, *p* = 0.0440, Figure 5B; for density of DCX^+^ cells: *r*^2^ = 0.3863, *p* = 0.0134, Figure 5C; for dendritic length of DCX^+^ cells: *r*^2^ = 0.3125, *p* = 0.0303, Figure 5D) but not with the time spent in the central area in the OFT (Figure 5F–H). After Bonferroni correction, the density of DCX^+^ cells still showed a significantly positive association with sucrose preference in the SPT. The data, which was consistent with previous reports, showed a significant association between reduced hippocampal neurogenesis and depressive behavior (i.e., the lower the level of hippocampal neurogenesis is, the more significant the depressive-like behavior), suggesting that the depression-like behavior of HFD-fed mice was related to neurogenesis impairment in the ventral hippocampus. Furthermore, a correlation between the Ki67^+^ cell density in the vSGZ and sucrose preference in the SPT (*r*^2^ = 0.6859, *p* = 0.0417, Figure 5B) was found before Bonferroni correction when only HFD-fed mice were included. Although this correlation seemed not significant after Bonferroni correction, it could prompt the tendency that the decline of hippocampal neurogenesis might contribute to the raised levels of depressive-like behavior in HFD-fed mice.

### 2.4. HFD Consumption in Adolescence Alters Microglial Morphology in the Ventral Hippocampus

**Figure 6:**
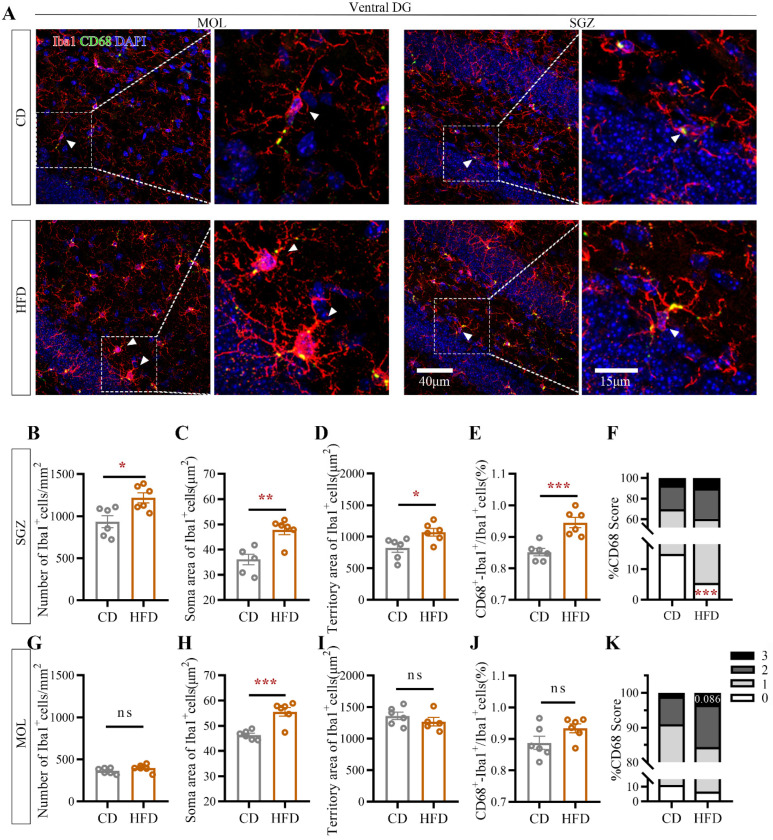
Effect of HFD consumption in adolescence on microglia in the mouse ventral hippocampus. (**A**) Fluorescence staining of Iba1^+^ and CD68^+^ cells in the ventral hippocampus of CD and HFD mice. Red, green, and blue fluorescence represent Iba1, CD68, and DAPI, respectively. The microglial density, average microglial soma area, average microglial territory area, percentage of CD68^+^ microglia, and percentage of CD68^+^ microglia with a score of 0 to 3 in the SGZ (**B**–**F**) and MOL (**G**–**K**) of the ventral hippocampus. The data are expressed as the mean ± SEM (*n* = 6 mice per group). When the HFD group was compared with the CD group, the data were analyzed using Student’s *t*-test. ns, not significant; * *p* < 0.05; ** *p* < 0.01; *** *p* < 0.001.

It is well established that there is a close association between microglial activity and neuronal plasticity in neurodevelopment and depression [30,31]. The characteristics of microglia in the molecular layer (MOL) and SGZ of the vDG, where neurogenesis impairment was the most pronounced in the above experiment, were evaluated by staining for the microglia-specific marker ionized calcium-binding adaptor molecule 1 (Iba1) and the phagocytic marker cluster of differentiation 68 (CD68, a widely used molecular marker for actively phagocytic microglia) (Figure 6A). Iba1 is expressed throughout the cytoplasm and thus outlines both the soma and processes of microglia [32]. Microglial morphology was visualized at a high resolution by fluorescence labeling followed by confocal microscopy. Consistent with previous reports, hippocampal microglia exhibited heterogeneity in a region-dependent manner [33]. Unlike those in CD-fed mice, microglia in HFD-fed mice were activated, as evidenced by significant increases in the cellular density in the vSGZ (Student’s *t*-test: *p* = 0.0124, Figure 6B), the soma area in the vSGZ (Student’s *t*-test: *p* = 0.0018, Figure 6C) and ventral MOL (vMOL, Student’s *t*-test: *p* = 0.0006, Figure 6H) and the territory area in the vSGZ (Student’s *t*-test: *p* = 0.0223, Figure 6D).

In addition, more microglia were positive for the lysosomal marker CD68 in HFD-fed mice than in CD-fed mice (in the vSGZ: Student’s *t*-test, *p* = 0.0008, Figure 6E). The expression of CD68 in microglia was enhanced in HFD-fed mice (in the vSGZ: for score 0: Student’s *t*-test, *p* = 0.0008, Figure 6F; in the vMOL: for score 3: Student’s *t*-test, *p* = 0.0864, Figure 6K), indicating excessive microglial activation in the vDG after HFD consumption in adolescence.

### 2.5. HFD-Fed Adolescent Mice Show Increased Microglial Engulfment of DCX^+^ Material in the Ventral Hippocampus

**Figure 7:**
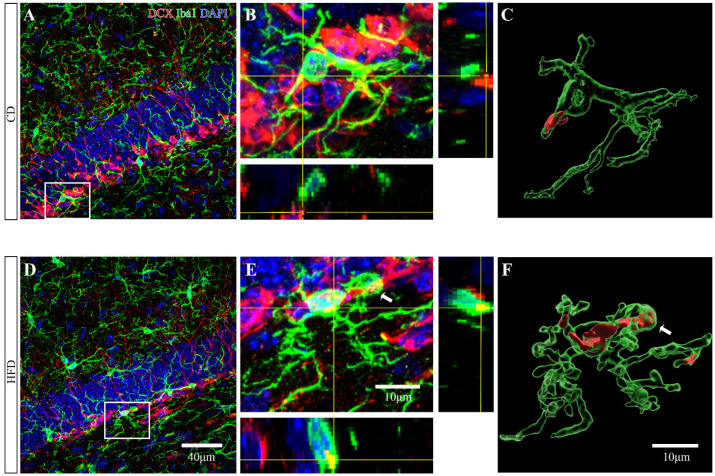
Effect of HFD consumption in adolescence on microglial phagocytosis of newborn neurons in the mouse ventral hippocampus. (**A**,**D**) High-resolution confocal images of Iba1^+^ and DCX^+^ cells in the ventral hippocampus of CD- and HFD-fed mice. Red, green, and blue fluorescence represent DCX, Iba1, and DAPI, respectively. (**B**,**E**) An orthogonal view of a confocal image showing the expression of DCX (red) within microglia (green). (**C**,**F**) Three-dimensional reconstruction and surface renderings were prepared using Imaris software (green and red surface renderings represent microglia and DCX staining inside). The white arrowheads indicate phagocytic cups of microglia.

**Figure 8:**
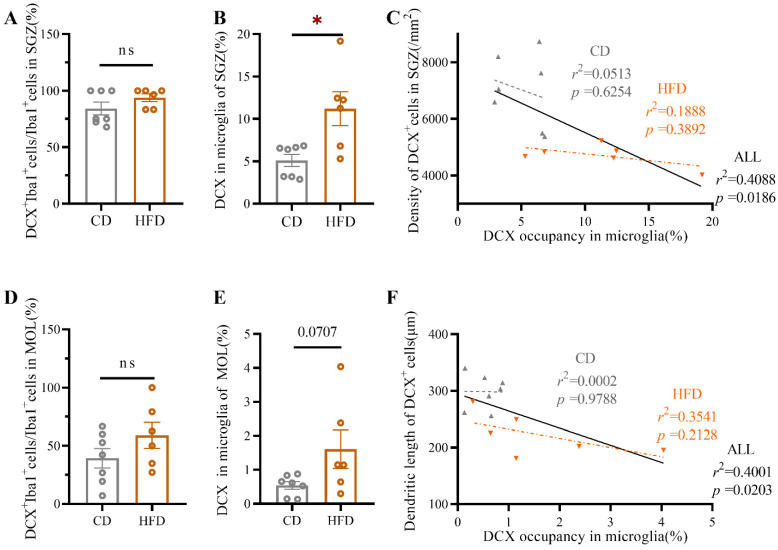
Quantification of the changes in microglial phagocytosis of newborn neurons in the mouse ventral hippocampus induced by HFD consumption in adolescence. (**A**,**D**) The percentage of DCX^+^Iba1^+^ cells among Iba1^+^ cells in the vSGZ and vMOL in CD- and HFD-fed mice. (**B**,**E**) Quantification of the percentage of the microglial volume occupied by DCX^+^ inclusions in the vSGZ and vMOL in CD- and HFD-fed mice. (**C**) Linear regression analysis of the microglial area occupied by DCX^+^ inclusions and the density of DCX^+^ cells in the vSGZ. (**F**) Linear regression analysis of the microglial area occupied by DCX^+^ inclusions and the total dendritic length of DCX^+^ cells in the vDG. Linear regressions are indicated by gray dotted lines (for CD-fed mice), orange dotted lines (for HFD-fed mice), and solid black lines (both CD and HFD-fed mice). The data are expressed as the mean ± SEM (*n* = 6–7 mice per group). When the HFD group was compared with the CD group, the data were analyzed using Student’s *t*-test. ns, not significant; * *p* < 0.05.

Newborn cells that are destined to become neurons and integrate into the neural network start to express the microtubule-associated protein DCX within several hours after genesis [34]. To determine whether excessive activation of microglia was involved in the atrophy of newborn neurons observed in HFD-fed mice, serial z-stack confocal images of DCX and Iba1 staining in the vDG were acquired (Figure 7A,B,D,E). Subsequently, we quantified the DCX^+^ material within the microglia in the vSGZ and vMOL by 3D surface volume rendering (Figure 7C,F). The percentage of DCX^+^ microglia was in both the vSGZ (Student’s *t*-test: *p* = 0.1895, Figure 8A) and vMOL (Student’s *t*-test: *p* = 0.1802, Figure 8D) comparable among groups. However, the percentage of the microglial volume occupied by DCX^+^ inclusions in the vSGZ was significantly increased in HFD-fed mice (Student’s *t*-test: *p* = 0.0109, Figure 8B). A similar trend was observed in the vMOL, but it did not reach significance (Student’s *t*-test: *p* = 0.0707, Figure 8E).

The association between microglial engulfment of DCX^+^ material and the density of DCX^+^ cells as well as the morphology of DCX^+^ cells was determined using linear regression analysis. As shown in Figure 8, significant correlations between DCX occupancy within microglia and the density of DCX^+^ cells in the vSGZ (*r*^2^ = 0.4088, *p* = 0.0186, Figure 8C) and between the DCX occupancy within microglia and the total dendritic length of DCX^+^ cells in the vDG (*r*^2^ = 0.4001, *p* = 0.0203, Figure 8F) were revealed when all mice were included, indicating that atrophy of newborn neurons was closely associated with increased engulfment of DCX^+^ material by excessively activated microglia.

Together, these findings illustrate that HFD consumption in adolescence drives newborn neuron atrophy through excessive microglial engulfment of DCX^+^ material.

## 3. Discussion

Recent evidence from epidemiological, clinical, and animal studies indicates that HFD consumption is associated with a high incidence of depression and anxiety [6,7,35,36,37,38]. Considering the increasing healthcare burden imposed by adolescent depression/anxiety [39,40] and the prevalence of HFD consumption among adolescents, it is imperative to explore the relationship between HFD consumption in adolescence and emotional disorders as well as the underlying neurobiological mechanism in animal models. The present study showed that HFD-fed adolescent mice exhibited elevated levels of depression and anxiety, impaired hippocampal neurogenesis, and excessive microglial activation in the ventral hippocampus. Notably, we found that the negative effects of HFD consumption in adolescence on emotion and neuroplasticity may be attributed at least in part to aberrant microglial engulfment of newborn neurons.

The hippocampus, a critical region in emotion regulation, is widely assumed to be particularly sensitive to HFD consumption [8,41,42], which may induce adverse changes in neuroplasticity [43,44,45,46,47,48]. Hippocampal neurogenesis, which describes the generation of new neurons that subsequently integrate into neural circuits in the DG, is a fundamental process associated with brain plasticity [49]. Decreased hippocampal neurogenesis has been identified as a common feature of some mood disorders [50,51]. Recent studies have also demonstrated that the efficacy of antidepressant and anti-anxiety drugs such as fluoxetine is dependent on successful neurogenesis in the ventral hippocampus [20,52], suggesting that neurogenesis in this brain region may be a key target of psychological risk factors. Studies by our group and others suggest that the ventral hippocampus plays a prominent role in emotional adjustment [53,54]. The results of our previous morphological analysis indicated that more prominent alterations occur in the ventral hippocampus after long-term HFD consumption and that these alterations are accompanied by abnormal emotional behavior in adult mice [54]. Similarly, Hui et al. found that the ventral hippocampus is a critical region involved in chronic stress-induced depression [55]. The level of hippocampal neurogenesis during adolescence is up to four times higher than that during adulthood, making alterations to neurogenesis during adolescence particularly consequential [23,56]. Consistent with previous reports linking hippocampal neurogenesis deficits with emotional perturbations induced by HFD consumption in adolescence [8,57], we found that the HFD-fed adolescent mice developed emotional abnormalities and exhibited reduced neurogenesis mainly in the ventral hippocampus. Furthermore, our data showed that the dendritic complexity of newborn neurons was decreased in the ventral hippocampi of HFD-fed mice, indicating that HFD consumption in adolescence interferes with the morphological maturation of new neurons and subsequently perturbs their integration into neuronal networks. Therefore, it is reasonable to speculate that impaired hippocampal neurogenesis, specifically in the ventral hippocampus, might play a critical role in mood disorders induced by HFD consumption in adolescence.

To verify this hypothesis, we analyzed the correlations between relevant indicators and significant changes in behavior and neurogenesis and found that only the proliferation and newborn neurons in the ventral hippocampus were significantly associated with emotional indicators, providing further evidence that impaired ventral hippocampal neurogenesis might be responsible for emotional dysfunction induced by HFD consumption in adolescence. Furthermore, depression-related behavior, but not anxiety-related behavior, was significantly associated with neurogenesis when all experimental subjects were included, suggesting that hippocampal neurogenesis impairment makes a greater contribution to HFD-related depression than HFD-related anxiety in adolescents. Interestingly, when only HFD-fed mice were considered, a correlation was found between the density of Ki67^+^ cells in the vSGZ and sucrose preference in the SPT before Bonferroni correction, suggesting the tendency that once cell proliferation falls below a certain level, the degree of depression might increase with the decline in cell proliferation.

Despite emerging reports showing that hippocampal neurogenesis is impaired after HFD consumption [8,57,58], the underlying neurobiological mechanisms remain to be identified. In recent years, the phenotype of neuroimmune-associated microglia in the hippocampus has been found to be altered after HFD consumption [59,60,61,62,63], suggesting that microglia appear to be involved in hippocampal neurogenesis deficits induced by HFD consumption [8,64]. After four months of HFD consumption, 5-month-old mice show increased levels of depression and anxiety and decreased hippocampal neurogenesis, as indicated by decreased cell proliferation and neuronal differentiation in the SGZ [8]. These phenomena are accompanied by neuroinflammatory, depicted by a reactive phenotype in microglia cells in the hippocampus [8]. In another study on the impact of early HFD consumption (postnatal day (P) 21–60) on the mouse hippocampus, neurogenic capability (density of ki67^+^ cells and DCX^+^ cells) was remarkably reduced in the SGZ, where microglial were activated, as evidenced by an increased number of microglia and an enlarged soma size [64].

Microglia, known as “brain gardeners,” have been found to influence neuronal proliferation, differentiation, and maturation [65,66,67]. Microglial morphology is closely linked to microglial functions [68,69], and changes in microglial phenotype are related to the impairment of hippocampal neurogenesis under pathological conditions [70]. In the healthy brain, microglia have a ramified morphology with a small soma and fine processes [68]. However, any pathological disturbance of brain homeostasis can evoke rapid and profound changes in microglial cell shape and function, defined as microglial activation [68,71,72]. Our data showed that microglial density, soma area, territory area, and CD68 expression in the ventral hippocampus were significantly increased in HFD-fed adolescent mice. Consistent with our results, it was found that in adult animals, the number and total process length of microglia in the hippocampal DG region are increased after HFD feeding for one month [73] and that microglia in these animals are distinct from traditional amoeba-like activated microglia [68]. Because microglial processes continually contact newborn neurons and play a functionally dynamic role in hippocampal neurogenesis, increases in microglial density, soma area, territory area, and CD68 expression in HFD-fed adolescent mice might indicate that the basal activity of microglia and their connections with adjacent cells are increased after HFD consumption in adolescence.

Ample evidence indicates that microglia play a multifaceted role in regulating neurogenesis, in turn influencing behavior [70,74]. In the hippocampus, neural progenitors in the SGZ give rise to mature neurons, but only a small subset of these cells integrate into the neural circuitry [32]. Sierra et al. found that the majority of newborn cells undergo apoptosis and are quickly cleared by microglia [32]. Under physiological conditions, this apoptosis-coupled phagocytosis of microglia is beneficial, preventing the spillage of detrimental cellular contents that can damage surrounding tissues from secondary necrotic cells and shaping hippocampal neurogenesis [32,75]. Microglia also eliminate redundant nascent neurons and aberrant newborn progenitor cells, thereby regulating the number of newborn cells that are incorporated into the functional circuits in animal models of status epilepticus [26]. However, aberrant microglia have been demonstrated to have deleterious effects on neurogenesis, especially in the hippocampus [27,76]. Alterations in microglia may contribute to a reduction in hippocampal neurogenesis and concomitant deficits in memory during neuroinflammation or aging or under other pathological conditions through the abnormal secretion of different factors (e.g., cytokines, trophic factors, etc.) [27]. In addition, microglia have been observed to promote abnormal hippocampal neurogenesis and an increase in the number of immature neuronal projections after seizures in epilepsy models [76].

In the present study, the DCX^+^ inclusions in microglia in the ventral hippocampus were increased in HFD-fed mice. Combined with our observations of the change in the expression of CD68 (a lysosomal marker indicative of phagocytic activity of microglia), this finding may indicate increased microglial engulfment of immature neurons. Regarding the effect of microglial phagocytosis on newborn neurons, the percentage between Iba1 (a microglial marker) and DCX colocalization was negatively correlated with the density and morphological complexity of DCX^+^ cells, further demonstrating a contributing role for excessive microglial phagocytosis of newborn neurons in the impairment of hippocampal neurogenesis after HFD consumption in adolescence. Decreases in the number and dendritic complexity of immature neurons induced by enhanced engulfment of microglia in HFD-fed mice may affect neuronal function and connectivity with other cells and, subsequently, the formation and functional dynamics of neuronal circuits and brain networks, confirming and expanding the existing understanding of the role of microglia in neurogenesis deficits, which lead to neuropsychological disorders. Nonetheless, our experiments only assessed the role of microglial phagocytosis in disrupting neurogenesis after early consumption of an HFD. Future studies on the effects of various microglial functions, such as secretion, are necessary to explore the underlying immune-related molecular mechanisms comprehensively. Meanwhile, studies to block microglial activation are imperative to determine a causative role for microglia in impaired hippocampal neurogenesis and affective dysregulation after HFD exposure in adolescence.

In summary, our findings show that HFD consumption in adolescence induces anxiety- and depression-like behavior and decreases hippocampal neurogenesis (especially in the ventral hippocampus) in mice. Furthermore, we propose for the first time that the adverse influence of HFD consumption in adolescence on behavior and hippocampal neurogenesis appears to be due to excessive phagocytosis of activated microglia. Given the lack of effective treatments for adolescent depression and anxiety and the high prevalence of HFD consumption in the adolescent population, the findings of the present study are important because they show that early consumption of an HFD leads to psychological disorder-related pathogenesis via excessive activation of hippocampal microglia and provide valuable experimental evidence for the development of glial-targeted therapies for emotional disorders.

## 4. Materials and Methods

### 4.1. Animals and Diets

Three-week-old male C57BL/6 mice were purchased from Hangzhou Ziyuan Laboratory Animal Science and Technology Co., Ltd. (Hangzhou, China, SCXK (ZHE) 2019-0004). All animals were housed in cages in groups (five to six animals per cage) in a temperature (22 ± 1 °C)- and humidity (55 ± 5%)-controlled room. They were maintained on a 12-h light/dark cycle (lights on at 7:00 a.m.) and provided ad libitum access to food and water. After one week of acclimatization, the mice were randomly assigned to either the HFD [energy density: 5.0 kcal/g; carbohydrate: 20.6%, fat: 60% (from a lard and soybean oil mixture containing a lard/soybean oil ratio of about 10:1), protein: 19.4%; diet TP23300] or CD (energy density: 3.5 kcal/g; carbohydrate: 70.6%, fat: 10%, protein: 19.4%; diet TP23303) group. The pellets fed to both groups were provided by Trophic Animal Feed High-Tech Co., Ltd. (Nantong, China). The adolescence period for mice ranges from weaning (about 3 weeks old) to adulthood (about 8 weeks old) [77,78,79]. In the present study, 4-week-old mice were fed for about 4 weeks, i.e., dietary exposure mostly throughout adolescence. One week before the behavioral test, the mice were habituated to handing by the experimenter. The behavioral experiments were performed on animals who were exposed to dietary treatment for 2 weeks (6 weeks old, the onset of puberty [80]; the SPT and OFT) or 4 weeks (8 weeks old, the end of adolescence; all behavioral tests) while they continued to consume their respective diet. Then, the animals were sacrificed after behavioral testing (Figure 1). All animal procedures were performed with approval by and in accordance with the University Committee for Laboratory Animals of Southeast University, China.

### 4.2. Behavioral Analysis

After dietary treatment for 2 weeks or 4 weeks, all mice underwent behavioral tests in the following order: the SPT, OFT, EZM, and FST. Considering the additional impact of each test on the animals, only the SPT and OFT were performed after 2 weeks of dietary treatment.

#### 4.2.1. SPT

Anhedonia (a core feature of clinical depression) was assessed by the SPT [54]. After habituation to two identical bottles containing water for 24 h, singly-housed mice were given two bottles: one containing water and the other containing 1% sucrose solution. During the test, the two bottles were switched every 12 h to reduce the effects of position preference. Twenty-four hours later, the bottles were weighed to determine the consumption of each fluid, and sucrose preference was calculated.

#### 4.2.2. FST

The FST was also performed to evaluate depressive-like behavior. The mice were placed in a transparent cylinder (diameter 13 cm; height 18.5 cm) filled with 24 ± 1 °C water to a depth of approximately 13 cm for 6 min and recorded using a video camera. The immobility time, defined as the duration for which a mouse stopped swimming and floated, making only those movements necessary to keep its head above water, was measured for the last 4 min.

#### 4.2.3. OFT

The OFT was used to assess depressive and anxiety-like behavior and locomotor activity. The test was performed in a 50 × 50 × 50 cm white opaque arena. After being gently placed in one corner of the arena, the mice were allowed to explore the arena for 5 min. Their behavior was automatically recorded with a video camera that was fixed above the arena and coupled with video-tracking software (visutrack 3.0, Xinruan Information Technology Company, Shanghai, China). We measured locomotor activity (expressed as the total distance traveled) and emotional behavior (expressed as the time spent in and the number of entries into the central area). The apparatus was cleaned with 70% ethanol and water after each session to remove odor cues.

#### 4.2.4. EZM

In the EZM test, the mice were placed at a randomly chosen boundary between an open and a closed zone facing the closed zone. An overhead camera linked to a computer with Shanghai Xinruan software (visutrack 3.0, Xinruan Information Technology Company, Shanghai, China) was used to track the position of each mouse for 5 min. The time spent in the closed zones of the mazes and the distance traveled were calculated. At the end of each session, the apparatus was cleaned with 70% ethanol and then water to remove odor cues.

### 4.3. Tissue Collection

The day after the FST, the animals were deeply anesthetized with pentobarbital (100 mg/kg, i.p.) between 09:00 and 12:00. As described in a previous report by our group [54], the mice were perfused transcardially with 20 mL of 0.1 M phosphate-buffered saline (PBS) followed by 20 mL of 4% paraformaldehyde (PFA) in 0.1 M PBS for immunohistochemistry. Then, the brains were quickly extracted and placed in 4% PFA for 6–8 h at 4 °C before being transferred to a 30% sucrose cryopreservation solution. When the brains sank, they were embedded in an optimal cutting temperature (OCT) compound and stored at −80 °C. Coronal sections of the dorsal (−0.9~−2.4 mm) and ventral (−2.4~−4.2 mm) hippocampus (40 µm thick) were prepared [81] for immunohistochemical staining.

### 4.4. Immunohistochemistry

Two to three bilateral brain sections (320 μm intervals) from each animal were evaluated to study neurogenesis or microglial characteristics. Both the ventral and dorsal hippocampus were evaluated unless specified otherwise. Free-floating sections were rinsed three times for 10 min in 0.1 M PBS and blocked with blocking solution before being incubated with primary antibodies overnight at 4 °C. The following day, the sections were washed six times for 10 min in 0.1 M PBS before being incubated for 2 h at room temperature with secondary antibodies diluted in blocking serum in a wet chamber away from light. The following antibodies were used: rabbit anti-Ki67 (for proliferating cells, 1:500, ab16667, Abcam, Cambridge, UK), guinea pig anti-DCX (for newly generated neurons, 1:1000, AB2253, Millipore, Temecula, CA, USA), rabbit anti-Iba1 (for microglia, 1:1000, 019-19741, Wako, Osaka, Japan), rat anti-CD68 (for phagocytic microglia, 1:1500, MCA1957, Bio-Rad, Oxford, UK), goat anti-Iba1 (for microglia, 1:600, 011-27991, Wako), Alexa Fluor 488-conjugated goat anti-rabbit IgG (1:1000, ab150077, Abcam), Alexa Fluor 568-conjugated goat anti-guinea pig IgG (1:1000, ab175714, Abcam), Alexa Fluor 594-conjugated donkey anti-rat IgG (1:1000, ab150156, Abcam), and Alexa Fluor 488-conjugated donkey anti-goat IgG (1:1000, ab150129, Abcam). All slides were counterstained with 4′6-diamidino-2-phenylindole (DAPI, 1:600, C1027, Beyotime, Shanghai, China) in PBS for 15 min to visualize the cell nuclei. For negative control sections, primary or secondary antibodies were omitted.

### 4.5. Quantitative Analysis of Immunohistochemical Data

Images were taken using the sequential acquisition of separate wavelength channels by confocal laser scanning microscopy (FV1000, FV3000, Olympus, Tokyo, Japan) under 40× and 60× objectives. The laser intensity of each channel was kept constant through the image acquisition period. All confocal stacks were acquired at a resolution of 1024 × 1024 pixels with a z-step of 1 μm. Samples were analyzed by an observer blinded to the experimental design using ImageJ software 1.52a (US National Institutes of Health, Bethesda, MD, USA) or Imaris software 9.8.0 (Bitplane, Oxford, UK).

For the analysis of the number of Ki67^+^ and DCX^+^ cells, only cells with cell bodies in the SGZ, defined as a layer of cells with a height of 20 μm (expanding 5 μm into the hilus and 15 μm into the granular cell layer) [32], were manually counted using the cell counter function of ImageJ. The morphological features of the DCX^+^ cells in the DG were also analyzed using ImageJ software. At least five individual cells in each animal from all experimental groups were randomly selected and reconstructed by tracing using the plugin NeuronJ. The total dendritic length, the total number of dendritic branches, and the number of dendritic intersections with concentric circles at radial intervals of 10 µm in Sholl analysis were analyzed to assess the complexity of the newborn neurons.

For the quantitative evaluation of microglia, only Iba1^+^ cells with a clearly visible cell body were analyzed. The microglial density, average territory area of microglia (defined as the area outlined by the outermost points of the dendritic processes of an Iba1^+^ cell), average microglial soma area, percentage of CD68^+^ microglia (defined as the proportion of CD68^+^Iba1^+^ cells among all Iba1^+^ cells) and percentage of CD68^+^ microglia in the ventral hippocampus with a microglial phagocytosis function score of 0 to 3 were analyzed as previously reported by our group and other laboratories [54,82,83]. A score of 0 indicated no/scarce CD68 staining, 1 signified punctate CD68 staining, 2 indicated CD68 staining covering one-third to two-thirds of the total area, and 3 signified a CD68 staining area greater than two-thirds of the total area.

For quantitative evaluation of microglial phagocytosis of newborn neurons, the percentage of DCX^+^Iba1^+^ cells among Iba1^+^ cells and the percentage of the microglial volume occupied by DCX^+^ inclusions in the SGZ and MOL of ventral hippocampus in CD- and HFD-fed adolescent mice were measured in 40-μm z-stacked confocal images (60× oil objective) by Imaris software.

### 4.6. Statistical Analysis

The data were plotted and analyzed statistically using GraphPad Prism 8 (GraphPad Software Inc., San Diego, CA, USA). All quantitative results are expressed as the mean ± standard error (SE). The significance of the difference between groups was evaluated using two-way ANOVA or two-tailed unpaired Student’s *t*-test as appropriate. Asterisks were used to indicate significance as follows: * *p* < 0.05, ** *p* < 0.01, *** *p* < 0.001. Values > 0.05 were considered not significant (ns).

## Data Availability

Data contained within the paper are available from the authors upon reasonable request.

## References

[1] Herrman H., Kieling C., McGorry P., Horton R., Sargent J., Patel V. (2019). Reducing the global burden of depression: A lancet—World psychiatric association commission. Lancet.

[2] Kessler R.C., Amminger G.P., Aguilar-Gaxiola S., Alonso J., Lee S., Üstün T.B. (2007). Age of onset of mental disorders: A review of recent literature. Curr. Opin. Psychiatry.

[3] Andersen S.L., Teicher M.H. (2008). Stress, sensitive periods and maturational events in adolescent depression. Trends Neurosci..

[4] Bernaras E., Jaureguizar J., Garaigordobil M. (2019). Child and adolescent depression: A review of theories, evaluation instruments, prevention programs, and treatments. Front. Psychol..

[5] Ljungberg T., Bondza E., Lethin C. (2020). Evidence of the importance of dietary habits regarding depressive symptoms and depression. Int. J. Environ. Res. Public Health.

[6] Masana M.F., Tyrovolas S., Kollia N., Chrysohoou C., Skoumas J., Haro J.M., Tousoulis D., Papageorgiou C., Pitsavos C., Panagiotakos D.B. (2019). Dietary patterns and their association with anxiety symptoms among older adults: The attica study. Nutrients.

[7] Jacka F.N., Mykletun A., Berk M., Bjelland I., Tell G.S. (2011). The association between habitual diet quality and the common mental disorders in community-dwelling adults: The hordaland health study. Psychosom. Med..

[8] Vinuesa A., Pomilio C., Menafra M., Bonaventura M.M., Garay L., Mercogliano M.F., Schillaci R., Lux Lantos V., Brites F., Beauquis J. (2016). Juvenile exposure to a high fat diet promotes behavioral and limbic alterations in the absence of obesity. Psychoneuroendocrinology.

[9] Li Y., Lv M.-R., Wei Y.-J., Sun L., Zhang J.-X., Zhang H.-G., Li B. (2017). Dietary patterns and depression risk: A meta-analysis. Psychiatry Res..

[10] Schneider M. (2013). Adolescence as a vulnerable period to alter rodent behavior. Cell Tissue Res..

[11] Larsen B., Luna B. (2018). Adolescence as a neurobiological critical period for the development of higher-order cognition. Neurosci. Biobehav. Rev..

[12] Spear L.P. (2013). Adolescent neurodevelopment. J. Adolesc. Health.

[13] Pascual M., López-Hidalgo R., Montagud-Romero S., Ureña-Peralta J.R., Rodríguez-Arias M., Guerri C. (2021). Role of mtor-regulated autophagy in spine pruning defects and memory impairments induced by binge-like ethanol treatment in adolescent mice. Brain Pathol..

[14] Hamilton J.P., Siemer M., Gotlib I.H. (2008). Amygdala volume in major depressive disorder: A meta-analysis of magnetic resonance imaging studies. Mol. Psychiatry.

[15] Sexton C.E., Mackay C.E., Ebmeier K.P. (2013). A systematic review and meta-analysis of magnetic resonance imaging studies in late-life depression. Am. J. Geriatr. Psychiatry.

[16] Sheline Y.I., Liston C., McEwen B.S. (2019). Parsing the hippocampus in depression: Chronic stress, hippocampal volume, and major depressive disorder. Biol. Psychiatry.

[17] Li Y., Luo Y., Tang J., Liang X., Wang J., Xiao Q., Zhu P., Xiao K., Jiang L., Dou X. (2021). The positive effects of running exercise on hippocampal astrocytes in a rat model of depression. Transl. Psychiatry.

[18] Lindqvist A., Mohapel P., Bouter B., Frielingsdorf H., Pizzo D., Brundin P., Erlanson-Albertsson C. (2006). High-fat diet impairs hippocampal neurogenesis in male rats. Eur. J. Neurol..

[19] Hill A.S., Sahay A., Hen R. (2015). Increasing adult hippocampal neurogenesis is sufficient to reduce anxiety and depression-like behaviors. Neuropsychopharmacology.

[20] Mahar I., Bambico F.R., Mechawar N., Nobrega J.N. (2014). Stress, serotonin, and hippocampal neurogenesis in relation to depression and antidepressant effects. Neurosci. Biobehav. Rev..

[21] Bassett B., Subramaniyam S., Fan Y., Varney S., Pan H., Carneiro A.M.D., Chung C.Y. (2021). Minocycline alleviates depression-like symptoms by rescuing decrease in neurogenesis in dorsal hippocampus via blocking microglia activation/phagocytosis. Brain Behav. Immun..

[22] Tanti A., Rainer Q., Minier F., Surget A., Belzung C. (2012). Differential environmental regulation of neurogenesis along the septo-temporal axis of the hippocampus. Neuropharmacology.

[23] Hueston C.M., Cryan J.F., Nolan Y.M. (2017). Stress and adolescent hippocampal neurogenesis: Diet and exercise as cognitive modulators. Transl. Psychiatry.

[24] Gomez-Nicola D., Perry V.H. (2015). Microglial dynamics and role in the healthy and diseased brain:A paradigm of functional plasticity. Neuroscientist.

[25] Paolicelli R.C., Bolasco G., Pagani F., Maggi L., Scianni M., Panzanelli P., Giustetto M., Ferreira T.A., Guiducci E., Dumas L. (2011). Synaptic pruning by microglia is necessary for normal brain development. Science.

[26] Luo C., Koyama R., Ikegaya Y. (2016). Microglia engulf viable newborn cells in the epileptic dentate gyrus. Glia.

[27] Rodríguez-Iglesias N., Sierra A., Valero J. (2019). Rewiring of memory circuits: Connecting adult newborn neurons with the help of microglia. Front. Cell Dev. Biol..

[28] Zhang J., Rong P., Zhang L., He H., Zhou T., Fan Y., Mo L., Zhao Q., Han Y., Li S. (2021). Il4-driven microglia modulate stress resilience through bdnf-dependent neurogenesis. Sci. Adv..

[29] Sato K. (2015). Effects of microglia on neurogenesis. Glia.

[30] Salter M.W., Stevens B. (2017). Microglia emerge as central players in brain disease. Nat. Med..

[31] Yirmiya R., Rimmerman N., Reshef R. (2015). Depression as a microglial disease. Trends Neurosci..

[32] Sierra A., Encinas J.M., Deudero J.J.P., Chancey J.H., Enikolopov G., Overstreet-Wadiche L.S., Tsirka S.E., Maletic-Savatic M. (2010). Microglia shape adult hippocampal neurogenesis through apoptosis-coupled phagocytosis. Cell Stem Cell.

[33] Spencer S.J., Basri B., Sominsky L., Soch A., Ayala M.T., Reineck P., Gibson B.C., Barrientos R.M. (2019). High-fat diet worsens the impact of aging on microglial function and morphology in a region-specific manner. Neurobiol. Aging.

[34] Brown J.P., Couillard-Després S., Cooper-Kuhn C.M., Winkler J., Aigner L., Kuhn H.G. (2003). Transient expression of doublecortin during adult neurogenesis. J. Comp. Neurol..

[35] Sanchez-Villegas A., Verberne L., De Irala J., Ruiz-Canela M., Toledo E., Serra-Majem L., Angel Martinez-Gonzalez M. (2011). Dietary fat intake and the risk of depression: The sun project. PLoS ONE.

[36] Hemmati A., Ghoreishy S.M., Karami K., Imani H., Farsani G.M., Mousavi S.E., Asoudeh F., Shariati-Bafghi S.-E., Karamati M. (2021). The association between dietary patterns and depression in adolescents: A cross-sectional study. Clin. Nutr. ESPEN.

[37] Yu H., Qin X., Yu Z., Chen Y., Tang L., Shan W. (2021). Effects of high-fat diet on the formation of depressive-like behavior in mice. Food Funct..

[38] Aucoin M., LaChance L., Naidoo U., Remy D., Shekdar T., Sayar N., Cardozo V., Rawana T., Chan I., Cooley K. (2021). Diet and anxiety: A scoping review. Nutrients.

[39] Bodden D.H.M., Stikkelbroek Y., Dirksen C.D. (2018). Societal burden of adolescent depression, an overview and cost-of-illness study. J. Affect. Disord..

[40] Yatham S., Sivathasan S., Yoon R., da Silva T.L., Ravindran A.V. (2018). Depression, anxiety, and post-traumatic stress disorder among youth in low and middle income countries: A review of prevalence and treatment interventions. Asian J. Psychiatry.

[41] Guillemot-Legris O., Muccioli G.G. (2017). Obesity-induced neuroinflammation: Beyond the hypothalamus. Trends Neurosci..

[42] de Paula G.C., Brunetta H.S., Engel D.F., Gaspar J.M., Velloso L.A., Engblom D., de Oliveira J., de Bem A.F. (2021). Hippocampal function is impaired by a short-term high-fat diet in mice: Increased blood–brain barrier permeability and neuroinflammation as triggering events. Front. Neurosci..

[43] Robison L.S., Albert N.M., Camargo L.A., Anderson B.M., Salinero A.E., Riccio D.A., Abi-Ghanem C., Gannon O.J., Zuloaga K.L. (2020). High-fat diet-induced obesity causes sex-specific deficits in adult hippocampal neurogenesis in mice. Eneuro.

[44] Arnold S.E., Lucki I., Brookshire B.R., Carlson G.C., Browne C.A., Kazi H., Bang S., Choi B.-R., Chen Y., McMullen M.F. (2014). High fat diet produces brain insulin resistance, synaptodendritic abnormalities and altered behavior in mice. Neurobiol. Dis..

[45] Wang X.-L., Kooijman S., Gao Y., Tzeplaeff L., Cosquer B., Milanova I., Wolff S.E.C., Korpel N., Champy M.-F., Petit-Demoulière B. (2021). Microglia-specific knock-down of bmal1 improves memory and protects mice from high fat diet-induced obesity. Mol. Psychiatry.

[46] Saiyasit N., Chunchai T., Apaijai N., Pratchayasakul W., Sripetchwandee J., Chattipakorn N., Chattipakorn S.C. (2020). Chronic high-fat diet consumption induces an alteration in plasma/brain neurotensin signaling, metabolic disturbance, systemic inflammation/oxidative stress, brain apoptosis, and dendritic spine loss. Neuropeptides.

[47] Dingess P.M., Darling R.A., Kurt Dolence E., Culver B.W., Brown T.E. (2017). Exposure to a diet high in fat attenuates dendritic spine density in the medial prefrontal cortex. Brain Struct. Funct..

[48] Rincel M., Lépinay A.L., Janthakhin Y., Soudain G., Yvon S., Da Silva S., Joffre C., Aubert A., Séré A., Layé S. (2018). Maternal high-fat diet and early life stress differentially modulate spine density and dendritic morphology in the medial prefrontal cortex of juvenile and adult rats. Brain Struct. Funct..

[49] Gonçalves J.T., Schafer S.T., Gage F.H. (2016). Adult neurogenesis in the hippocampus: From stem cells to behavior. Cell.

[50] Abrous D.N., Koehl M., Lemoine M. (2022). A baldwin interpretation of adult hippocampal neurogenesis: From functional relevance to physiopathology. Mol. Psychiatry.

[51] Sahay A., Hen R. (2007). Adult hippocampal neurogenesis in depression. Nat. Neurosci..

[52] Levone B.R., Cryan J.F., O’Leary O.F. (2015). Role of adult hippocampal neurogenesis in stress resilience. Neurobiol. Stress.

[53] Tannenholz L., Jimenez J.C., Kheirbek M.A. (2014). Local and regional heterogeneity underlying hippocampal modulation of cognition and mood. Front. Behav. Neurosci..

[54] Zhuang H., Yao X., Li H., Li Q., Yang C., Wang C., Xu D., Xiao Y., Gao Y., Gao J. (2022). Long-term high-fat diet consumption by mice throughout adulthood induces neurobehavioral alterations and hippocampal neuronal remodeling accompanied by augmented microglial lipid accumulation. Brain Behav. Immun..

[55] Ma H., Li C., Wang J., Zhang X., Li M., Zhang R., Huang Z., Zhang Y. (2021). Amygdala-hippocampal innervation modulates stress-induced depressive-like behaviors through ampa receptors. Proc. Nal. Acad. Sci. USA.

[56] He J., Crews F.T. (2007). Neurogenesis decreases during brain maturation from adolescence to adulthood. Pharmacol. Biochem. Behav..

[57] Lama A., Pirozzi C., Annunziata C., Morgese M.G., Senzacqua M., Severi I., Calignano A., Trabace L., Giordano A., Meli R. (2021). Palmitoylethanolamide counteracts brain fog improving depressive-like behaviour in obese mice: Possible role of synaptic plasticity and neurogenesis. Br. J. Pharmacol..

[58] Boitard C., Etchamendy N., Sauvant J., Aubert A., Tronel S., Marighetto A., Layé S., Ferreira G. (2012). Juvenile, but not adult exposure to high-fat diet impairs relational memory and hippocampal neurogenesis in mice. Hippocampus.

[59] Carey A.N., Gildawie K.R., Rovnak A., Thangthaeng N., Fisher D.R., Shukitt-Hale B. (2019). Blueberry supplementation attenuates microglia activation and increases neuroplasticity in mice consuming a high-fat diet. Nutr. Neurosci..

[60] Wu M., Liao M., Huang R., Chen C., Tian T., Wang H., Li J., Li J., Sun Y., Wu C. (2022). Hippocampal overexpression of trem2 ameliorates high fat diet induced cognitive impairment and modulates phenotypic polarization of the microglia. Genes Dis..

[61] Saiyasit N., Chunchai T., Prus D., Suparan K., Pittayapong P., Apaijai N., Pratchayasakul W., Sripetchwandee J., Chattipakorn M.D.P.D.N., Chattipakorn S.C. (2020). Gut dysbiosis develops before metabolic disturbance and cognitive decline in high-fat diet–induced obese condition. Nutrition.

[62] Kang E.-B., Koo J.-H., Jang Y.-C., Yang C.-H., Lee Y., Cosio-Lima L.M., Cho J.-Y. (2016). Neuroprotective effects of endurance exercise against high-fat diet-induced hippocampal neuroinflammation. J. Neuroendocrinol..

[63] Butler M.J., Cole R.M., Deems N.P., Belury M.A., Barrientos R.M. (2020). Fatty food, fatty acids, and microglial priming in the adult and aged hippocampus and amygdala. Brain Behav. Immun..

[64] Vinuesa A., Bentivegna M., Calfa G., Filipello F., Pomilio C., Bonaventura M.M., Lux-Lantos V., Matzkin M.E., Gregosa A., Presa J. (2019). Early exposure to a high-fat diet impacts on hippocampal plasticity: Implication of microglia-derived exosome-like extracellular vesicles. Mol. Neurobiol..

[65] Pérez-Rodríguez D.R., Blanco-Luquin I., Mendioroz M. (2021). The participation of microglia in neurogenesis: A review. Brain Sci..

[66] De Lucia C., Rinchon A., Olmos-Alonso A., Riecken K., Fehse B., Boche D., Perry V.H., Gomez-Nicola D. (2016). Microglia regulate hippocampal neurogenesis during chronic neurodegeneration. Brain Behav. Immun..

[67] Gemma C., Bachstetter A. (2013). The role of microglia in adult hippocampal neurogenesis. Front. Cell. Neurosci..

[68] Kettenmann H., Hanisch U.-K., Noda M., Verkhratsky A. (2011). Physiology of microglia. Physiol. Rev..

[69] Liu T., Lu J., Lukasiewicz K., Pan B., Zuo Y. (2021). Stress induces microglia-associated synaptic circuit alterations in the dorsomedial prefrontal cortex. Neurobiol. Stress.

[70] Al-Onaizi M., Al-Khalifah A., Qasem D., ElAli A. (2020). Role of microglia in modulating adult neurogenesis in health and neurodegeneration. Int. J. Mol. Sci..

[71] Marzan D.E., Brügger-Verdon V., West B.L., Liddelow S., Samanta J., Salzer J.L. (2021). Activated microglia drive demyelination via csf1r signaling. Glia.

[72] Minaya D.M., Turlej A., Joshi A., Nagy T., Weinstein N., DiLorenzo P., Hajnal A., Czaja K. (2020). Consumption of a high energy density diet triggers microbiota dysbiosis, hepatic lipidosis, and microglia activation in the nucleus of the solitary tract in rats. Nutr. Diabetes.

[73] Li Y., Cheng Y., Zhou Y., Du H., Zhang C., Zhao Z., Chen Y., Zhou Z., Mei J., Wu W. (2022). High fat diet-induced obesity leads to depressive and anxiety-like behaviors in mice via ampk/mtor-mediated autophagy. Exp. Neurol..

[74] Vega-Rivera N.M., Ortiz-López L., Granados-Juárez A., Estrada-Camarena E.M., Ramírez-Rodríguez G.B. (2020). Melatonin reverses the depression-associated behaviour and regulates microglia, fractalkine expression and neurogenesis in adult mice exposed to chronic mild stress. Neuroscience.

[75] Abiega O., Beccari S., Diaz-Aparicio I., Nadjar A., Layé S., Leyrolle Q., Gómez-Nicola D., Domercq M., Pérez-Samartín A., Sánchez-Zafra V. (2016). Neuronal hyperactivity disturbs atp microgradients, impairs microglial motility, and reduces phagocytic receptor expression triggering apoptosis/microglial phagocytosis uncoupling. PLoS Biol..

[76] Mo M., Eyo U.B., Xie M., Peng J., Bosco D.B., Umpierre A.D., Zhu X., Tian D.-S., Xu P., Wu L.-J. (2019). Microglial p2y12 receptor regulates seizure-induced neurogenesis and immature neuronal projections. J. Neurosci..

[77] Brust V., Schindler P.M., Lewejohann L. (2015). Lifetime development of behavioural phenotype in the house mouse (mus musculus). Front. Zool..

[78] Laviola G., Macrì S., Morley-Fletcher S., Adriani W. (2003). Risk-taking behavior in adolescent mice: Psychobiological determinants and early epigenetic influence. Neurosci. Biobehav. Rev..

[79] Spear L.P. (2000). The adolescent brain and age-related behavioral manifestations. Neurosci. Biobehav. Rev..

[80] Dutta S., Sengupta P. (2016). Men and mice: Relating their ages. Life Sci..

[81] Lehmann M.L., Brachman R.A., Martinowich K., Schloesser R.J., Herkenham M. (2013). Glucocorticoids orchestrate divergent effects on mood through adult neurogenesis. J. Neurosci..

[82] Hong S., Beja-Glasser V.F., Nfonoyim B.M., Frouin A., Li S., Ramakrishnan S., Merry K.M., Shi Q., Rosenthal A., Barres B.A. (2016). Complement and microglia mediate early synapse loss in alzheimer mouse models. Science.

[83] Kreisel T., Frank M.G., Licht T., Reshef R., Ben-Menachem-Zidon O., Baratta M.V., Maier S.F., Yirmiya R. (2014). Dynamic microglial alterations underlie stress-induced depressive-like behavior and suppressed neurogenesis. Mol. Psychiatry.

